# Quantitative Susceptibility Mapping Shows Size-dependent Focused Ultrasound–mediated Therapeutic Delivery to Naive Brain and Gliomas in Mice

**DOI:** 10.1148/radiol.251005

**Published:** 2026-07-21

**Authors:** Matthew R. Hoch, Victoria R. Breza, G. Wilson Miller, Richard J. Price

**Affiliations:** ^1^Department of Biomedical Engineering, University of Virginia, 415 Lane Rd, MR-5, Rm 2316, Charlottesville, VA 22908; ^2^Department of Radiology & Medical Imaging, University of Virginia, Charlottesville, Va

## Abstract

**Background:**

Focused ultrasound US (FUS)–mediated disruption of the blood-brain barrier (BBB) and blood-tumor barrier (BTB) augments therapeutic delivery; however, relationships between dose, agent size, and delivery to both healthy and tumor tissue with FUS remain poorly defined.

**Purpose:**

To evaluate quantitative susceptibility mapping (QSM) for quantifying delivery of iron oxide nanoparticles (IONPs) after FUS-mediated BBB and BTB disruption and investigate how delivery efficiency varies with particle size in naive and glioma-bearing mice.

**Materials and Methods:**

FUS-mediated BBB and BTB disruption was performed on 8–11-week-old naive (*n* = 31) or GL261 glioma-bearing (*n* = 26) female C57BL/6 mice using albumin-shelled microbubbles and acoustic feedback control. Four contrast agents were delivered: 2.3-nm gadobenate dimeglumine and 15-, 23-, and 45-nm IONPs. Multi-echo gradient-echo MRI scans acquired before and after FUS were reconstructed into susceptibility maps and then converted to concentration maps. Tumor measurements were performed for 2.3- and 45-nm agents.

**Results:**

FUS-mediated delivery to naive brain varied with contrast agent size. Compared with 2.3-nm gadobenate dimeglumine (mean, 0.007 percentage injected dose [%ID] ± 0.001 [SD]), delivery increased 2.6-fold at 15 nm (mean, 0.017 %ID ± 0.002; *P* = .008) and was similar at 23 nm (mean, 0.015 %ID ± 0.002; *P* = .94) but decreased 2.5-fold at 45 nm (mean, 0.006 %ID ± 0.001; *P* =.004) relative to 23 nm. In gliomas, FUS increased delivery 1.9-fold for gadobenate dimeglumine (mean, 0.56 %ID/g ± 0.07 vs 1.08 %ID/g ± 0.16; *P* = .032) and 3.6-fold for 45-nm IONPs (mean, 0.39 %ID/g ± 0.06 vs 1.41 %ID/g ± 0.32; *P* = .001). Compared with naive brain, glioma delivery was similar for both gadobenate dimeglumine (mean, 0.87 %ID/g ± 0.06 vs 1.08 %ID/g ± 0.16; *P* = .26) and for 45-nm IONPs (mean, 1.76 %ID/g ± 0.34 vs 1.41 %ID/g ± 0.32; *P* = .47).

**Conclusion:**

QSM after MRI and FUS revealed that FUS-mediated delivery of contrast agents of therapeutically relevant sizes to naive murine brain varied with contrast agent size. Delivery to gliomas was similar to that to naive brain.

*Supplemental material is available for this article.*

© The Author(s) 2026. Published by the Radiological Society of North America under a CC BY 4.0 license.

## Graphical abstract

**Figure:**
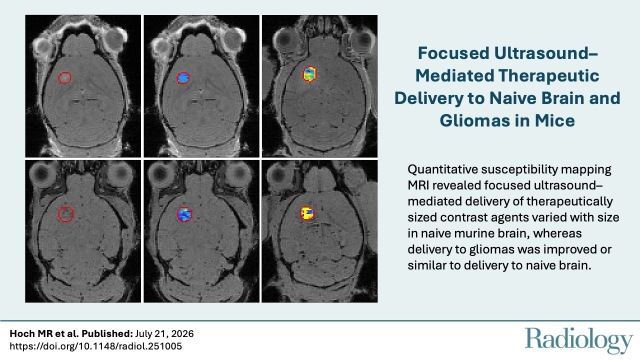

**Summary**

Quantitative susceptibility mapping MRI revealed focused ultrasound–mediated delivery of therapeutically sized contrast agents varied with size in naive murine brain, whereas delivery to gliomas was improved or similar to delivery to naive brain.

**Key Results**

■ Quantitative susceptibility mapping MRI revealed that focused ultrasound (FUS)–mediated delivery of contrast agents to naive murine brain varied with size, where the percentage injected dose [%ID] delivered increased 2.6-fold from 2.3 nm to 15 nm (*P* = .008) but decreased 2.5-fold from 23 nm to 45 nm (*P* = .004).
■ FUS enhanced brain tumor delivery by 1.9-fold for 2.3-nm gadobenate dimeglumine (%ID/g delivered: 0.56 ± 0.07 vs 1.08 ± 0.16; *P* = .032) and 3.6-fold for 45-nm iron oxide nanoparticles (IONPs) (0.39 ± 0.06 vs 1.41 ± 0.32; *P* = .001).
■ Compared with delivery to naive brain, FUS-mediated delivery to gliomas was similar for both 2.3-nm gadobenate dimeglumine (*P* = .26) and 45-nm IONPs (*P* = .47).

## Introduction

The blood-brain barrier (BBB) protects the central nervous system from blood-borne solutes and pathogens. Although essential for brain health and homeostasis, it also restricts the delivery of most drugs larger than 400–500 Da (1). This challenge is further compounded in brain tumors by the blood-tumor barrier (BTB), which is characterized by increased interstitial pressure, heterogeneous perfusion, and variable vascular permeability (2). Focused ultrasound (FUS), when combined with intravenously administered microbubbles, overcomes these transport limitations. FUS induces volumetric oscillations of the intravascular microbubbles, leading to a localized and transient disruption of the BBB and BTB. This disruption subsequently enables the delivery of therapeutics that would otherwise be severely limited or completely obstructed.

Neurotherapeutics delivered via FUS-mediated BBB and BTB disruption—including chemotherapies, antibodies, and viral or nonviral gene vectors—span a broad range of hydrodynamic diameters. Therefore, a precise understanding of how therapeutic size influences delivery is essential. Such insight would aid in selecting appropriate agents, optimizing treatment planning, and assessing efficacy in both preclinical and clinical settings. It could also inform the design of future therapeutics engineered for improved delivery and, ultimately, better patient outcomes.

Recent studies have used various imaging techniques, such as fluorescence, two-photon microscopy, PET/CT, and MRI (with T1 or T2 mapping), to investigate such size-dependent relationships (3–13). Although these studies generally suggest that delivery decreases with increasing molecular size, the evidence is often derived from qualitative fluorescence imaging or ex vivo bulk tissue measurements that lack spatial resolution and mainly focus on healthy brain tissue. Quantitative techniques, such as PET/CT and MRI, face their own limitations: PET/CT is constrained by low spatial resolution, T1 mapping is limited by the availability of contrast agents with therapeutically relevant sizes, T2 mapping is limited by long acquisitions, and both T1 and T2 mapping have reduced sensitivity at low concentrations of contrast agents. Furthermore, contrast agent relaxivities change in vivo, leading to potential errors in quantification (14). Collectively, these challenges have hindered efforts to precisely define how hydrodynamic diameter influences delivery in the context of FUS-mediated BBB and BTB disruption.

To overcome these imaging limitations, we propose a novel application of quantitative susceptibility mapping (QSM)—an advanced postprocessing MRI technique that derives tissue magnetic susceptibility from the phase evolution of multi-echo gradient-recalled echo data. QSM has been widely applied in neurologic research to quantify iron deposition, detect calcifications and hemorrhages, and enhance structural brain imaging—including water and/or myelin mapping relevant to such diseases as multiple sclerosis (15–17).

In this study, we aimed to evaluate QSM in quantifying the delivery of superparamagnetic iron oxide nanoparticles (IONPs) to the brain after FUS-mediated BBB and BTB disruption and investigate how delivery efficiency varies with particle size in both naive and glioma-bearing mice.

## Materials and Methods

### Study Design

In this animal study, experiments were approved by the Animal Care and Use Committee at the University of Virginia and conformed to the National Institutes of Health guidelines for the use of animals in research. Experiments were conducted between October 2023 and December 2025. All data generated or analyzed during the study are included in the published paper. All MRI scans are publicly accessible at *https://doi.org/10.18130/V3/QGVZHW*.

### Intracranial Tumor Cell Inoculation

GL261-Luc 2 (GL261) tumors were implanted into C57BL/6 mice as described in Appendix S1.

### Characterization and Preparation of Contrast Agents

Carboxyl-coated IONPs with iron cores of 5-, 10-, and 30-nm nominal diameter were purchased from Ocean NanoTech. Mean hydrodynamic diameter information provided by the vendor was used in the study analysis. To characterize particles of smaller size, MultiHance (Bracco Diagnostics; generic name: gadobenate dimeglumine), a gadolinium-based contrast agent with hydrodynamic size well established at 2.3 nm (18), was also used as a susceptibility contrast agent. QSM calibration curves for each contrast agent were generated by measuring the parts per million shift at five different concentrations. For IONPs, concentrations of 0, 25, 50, 75, and 100 µg Fe/mL were tested; concentrations of 0, 0.25, 0.5, 1, and 2.0 mmol/L were used for MultiHance. All solutions were prepared in 0.9% saline, placed into plastic vials, and submerged in a 0.9% saline bath.

### MRI-guided FUS Procedures

The FUS procedure was conducted under MRI guidance using the RK-300 small-bore FUS device (FUS Instruments). For the naive brain treatments, 8–11-week-old female C57BL/6 mice (*n* = 31) were anesthetized with an intraperitoneal injection of ketamine (50 mg/kg; Zoetis) and dexdomitor (0.25 mg/kg; Pfizer) in sterilized 0.9% saline and prepared by shaving and depilating their heads before being placed in a supine position and coupled to the transducer using degassed US gel. FUS targeting consisted of a single sonication point in the left or right striatum, and a BBB opening was achieved using a 1.15-MHz single-element transducer with a 10-msec burst length over a 1500-msec period. A total of 60 sonications were administered during a 1.5-minute sonication duration.

Aureus software (FUS Instruments), operating in the “Blood-Brain Barrier” mode, facilitated passive cavitation detection–modulated peak negative pressure. The feedback control system parameters were set as follows: a starting pressure of 0.375 MPa, pressure increment of 0.05 MPa, maximum pressure of 0.45 MPa, 20 sonication baselines without microbubbles, area under the curve (AUC) bandwidth of 500 Hz, AUC threshold of 10 SDs, pressure drop of 0.95, and frequency selection of the subharmonic, first ultraharmonic, and second ultraharmonic. In this context, AUC refers to the area under the curve of the frequency spectrum, and the specified bandwidth defines the range around each harmonic frequency over which the spectral power is integrated. Optison (GE HealthCare) microbubbles were intravenously injected as a bolus dose of 2 × 10^5^ microbubbles per gram of body weight. Contrast agents were intravenously injected immediately before the microbubbles with the dosing regimen shown in Table 1 (19).

**Table 1:** Contrast Agent Dosing for Focused Ultrasound Experiments

Contrast Agent	Iron Dosing (mg Fe/kg Body Weight)	Particle Amount (nmol/mg Fe)	Dosing (nmol/kg)
MultiHance	NA	NA	500 000
5-nm core IONP	25	6.9	172.5
10-nm core IONP	25	0.86	21.5
30-nm core IONP	25	0.034	0.85

Note.—IONP = iron oxide nanoparticle, NA = not applicable.

For tumor-bearing mice, all parameters remained the same except that an appropriate number of sonication spots was used to cover the entire tumor volume. This typically ranged from five to seven total sonication spots. Experimental groups for the naive brain and brain tumor studies are shown in Table 2.

**Table 2:** Experimental Groups for FUS-mediated Delivery Studies to Naive and Tumor-bearing Mice

Variable	Naive Brain (*n* = 31)					Tumor Bearing (*n* = 26)			
	Seven Mice	Four Mice	Eight Mice	Seven Mice	Five Mice	Eleven Mice	Six Mice	Five Mice	Four Mice
FUS	+	+	+	+	+	−	+	−	+
MultiHance	+	−	−	−	−	+	+	−	−
5-nm core IONP	−	+	−	−	−	−	−	−	−
10-nm core IONP	−	−	+	−	−	−	−	−	−
30-nm core IONP	−	−	−	+	−	−	−	+	+

Note.—Plus sign indicates that experimental group received treatment, negative sign indicates that treatment is not relevant to experimental group. Neither 5-nm nor 10-nm core iron oxide nanoparticles (IONPs) were tested in tumor-bearing mice. FUS = focused ultrasound.

### MRI Procedures

**Figure 1:**
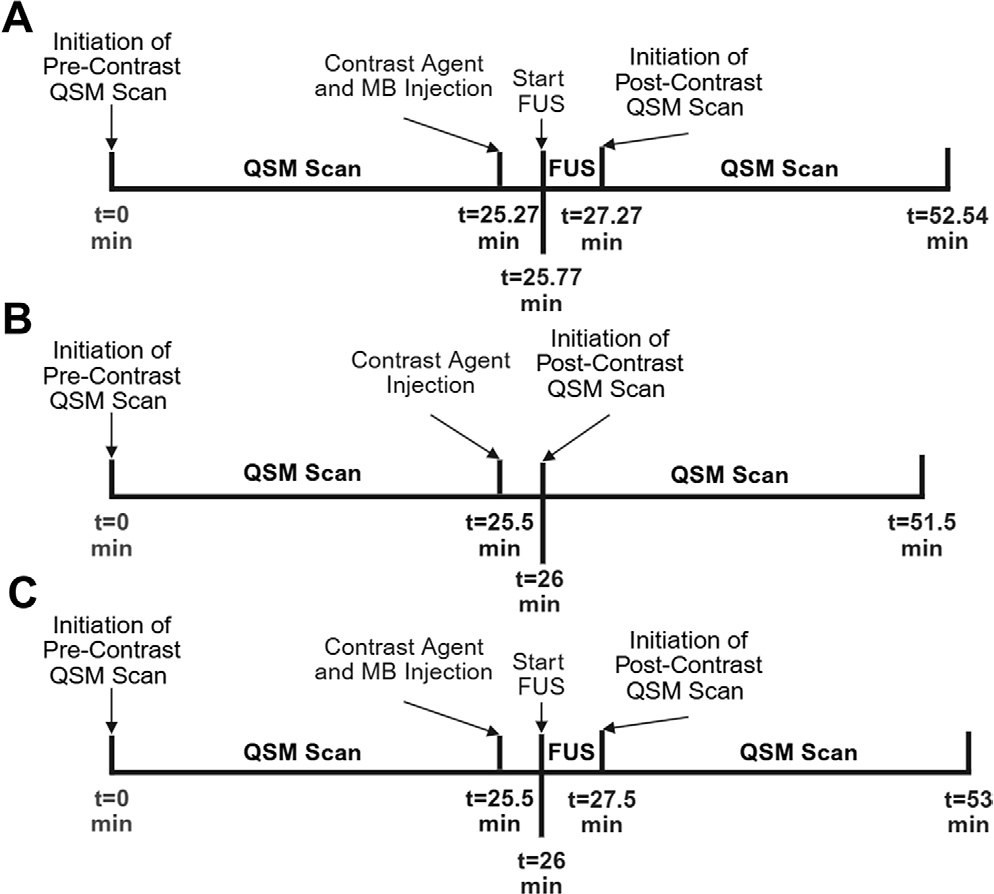
Experimental timelines for all in vivo focused ultrasound (FUS) experiments with delivery characterization by quantitative susceptibility mapping (QSM). **(A)** Timeline for the experiments involving naive murine brain, corresponding to Figures 3 and 4. At t = 0 minutes, precontrast QSM scanning begins. At 25.27 minutes, microbubbles (MB) and contrast agents are intravenously injected. At 25.77 minutes, FUS treatment is initiated. At 27.27 minutes, post-FUS QSM scanning is initiated. **(B)** Timeline depicts baseline blood-tumor barrier (BTB) measurements on day 13 or 14 after inoculation, corresponding to Figure 5. At t = 0 minutes, precontrast QSM scanning begins. At 25.5 minutes, contrast agent is intravenously injected. At 26 minutes, postcontrast QSM scanning is initiated. **(C)** Timeline depicts BTB disruption measurements on day 13 or 14 after inoculation, also corresponding to Figure 5. At t = 0 minutes, precontrast QSM scanning begins. At 25.5 minutes, microbubbles and contrast agents are intravenously injected. At 26 minutes, FUS treatment is initiated. At 27.5 minutes, post-FUS QSM scanning is initiated.

Multiecho gradient-echo images were acquired before administration of contrast agent and FUS to establish a baseline; images were also acquired after the FUS procedure for a postdelivery measurement. All images were acquired with a 9.4-T Bruker scanner (Bruker 94/20USR) with motion averaging enabled. The imaging parameters for the naive and tumor mouse studies are shown in Table 3. Figure 1 provides a schematic timeline for these experiments.

**Table 3:** Imaging Parameters for Pre- and Post-FUS Multi-Echo MRI for Quantitative Susceptibility Mapping in Mice

Group	TR (msec)	Flip Angle (degrees)	TE (msec)	FOV (mm)	Resolution (mm)	No. of Signal Averages	Acquisition Time (min)
Phantom (*n* = 4)	13.85	8	1.58, 2.89, 4.20, 5.52, 6.83, 8.14, 9.45, 10.77	52 × 52 × 40.04	0.260 × 0.260 × 0.260	5	35.56
Naive (*n* = 31)	22.01	9	3.21, 5.07, 6.94, 8.80, 10.66, 12.53, 14.39, 16.25	22 × 26.2 × 12.81	0.156 × 0.156 × 0.156	5	25.27
Tumor (*n* = 26)	22.32	9	3.21, 5.17, 7.13, 9.09, 11.05, 13.01, 14.97, 16.93	22 × 26.2 × 15.92	0.156 × 0.156 × 0.156	4	25.50

Note.—Table contains imaging parameters for MRI scans for eventual conversion to quantitative susceptibility maps. Phantom groups were needed to establish molar or mass magnetic susceptibilities for contrast agents. Naive groups investigated the effect of molecular size on focused ultrasound (FUS)–mediated delivery to healthy brain tissue. Tumor groups investigated the effect of molecular size on FUS-mediated delivery to tumor tissue as well as how the tumor microenvironment affects delivery relative to healthy brain tissue. FOV = field of view, TE = echo time, TR = repetition time.

### Imaging Analysis

Quantitative susceptibility maps were generated in MATLAB R2023a (MathWorks) using the Sepia Toolbox. Briefly, echo phase combination was performed with Morphology Enabled Dipole Inversion, or MEDI, nonlinear fit, and phase unwrapping was performed with GraphCut (20–22). Background field removal was performed with the projection onto dipole fields, or PDF, algorithm (23), and the dipole inversion step was performed with the Iterative Least Squares QR, or iLSQR, algorithm using a lambda value of 0.2 (24).

IONP concentration per voxel was calculated using Equation 1, in which *C_IONP_* is the IONP concentration (in nanomoles), *C_NP_* (measured in nanomoles per milligram of iron) is the nanoparticle nanomolar conversion factor shown in Table 2, *χ_Pre_* and *χ_Post_* are tissue susceptibilities before and after FUS, and *χ_Fe_* is the mass susceptibility of iron for the respective IONP (measured in parts per million times milliliter per milligram of iron), determined by the slope of the calibration curve for the respective particle, as follows:


(1) $$C_{IONP}=\frac{\left(\chi_{Post}-\chi_{\mathrm{Pr}e}\right)}{\chi_{Fe}}\cdot \;C_{NP}*1000.$$

A similar approach was implemented for the MultiHance quantification, except that the denominator was changed to the molar susceptibility of gadolinium, determined by the slope of the calibration curve for MultiHance.

The naive brain percentage injected dose (%ID) data were calculated using Equation 2, in which *V* represents the voxel volume (measured in liters), ROI represents the region of interest, and ID is the injected dose (measured in nanomoles) of the respective particle, as follows:


(2) $$\%ID\;=\;\left(\frac{\sum_{ROI}\left(C_{IONP/Multi}*V\right)}{ID}\right)\times 100.$$

The tumor percentage injected dose per gram data were calculated using Equation 3, in which *V *represents the voxel volume (measured in liters), ID is the injected dose (measured in nanomoles) of the respective particle, and ρ is the density of brain tissue (measured in grams per liter) (25), as follows:


(3) %IDg = ((CIONP/Multi*V)ID (ρ⋅V))×100.

The %ID/g values were calculated for each respective voxel within the tumor region of interest and subsequently averaged to produce the presented results.

### Region of Interest Selection

Regions of interest were selected as described in Appendix S2.

### Statistical Analysis

All results are presented as means ± SEMs. Statistical significance was set at *P* < .05, and analyses were performed by an author (M.R.H.) using Prism software, version 10.4.1 (GraphPad). Calibration curve data were fitted to a linear regression model using least-squares analysis, and the coefficient of determination (*R*^2^) was used to assess goodness of fit. Comparisons of average susceptibility change among nanoparticle-negative, FUS-positive and/or nanoparticle-negative, and FUS-positive and MultiHance groups were performed using one-way analysis of variance with the Dunnett multiple comparison test. Comparisons between contralateral and FUS-treated average susceptibility changes for all four contrast agents were analyzed with two-way analysis of variance (main effects only) using the Sidak multiple comparison test. Comparisons of %ID delivered among all four contrast agents were performed using one-way analysis of variance with the Tukey multiple comparison test. A robust regression and outlier removal test with a Q value of 1% was performed on the %ID comparisons. The %ID/g delivered for MultiHance and 45-nm IONP at baseline and after FUS was analyzed with two-way analysis of variance with the Tukey multiple comparison test. Comparisons of %ID/g delivered between naive brain and tumor groups for MultiHance and 45-nm IONP were conducted using unpaired *t* tests with Welch correction. Comparisons of fold change in brain tumor delivery over average baseline between MultiHance and 45-nm IONP were conducted using a one-tailed unpaired *t* test with Welch correction, testing the hypothesis that fold change was greater for the larger particles given that larger particles are challenged to enter brain tumors at baseline (26). Comparisons of baseline leakiness of MultiHance and 45-nm IONPs were conducted using unpaired *t* tests with Welch correction. Post hoc power analyses were run on all datasets. Power was greater than 0.8 for all datasets.

## Results

### Tissue Susceptibility Changes in Response to FUS-mediated Contrast Agent Delivery

**Figure 2:**
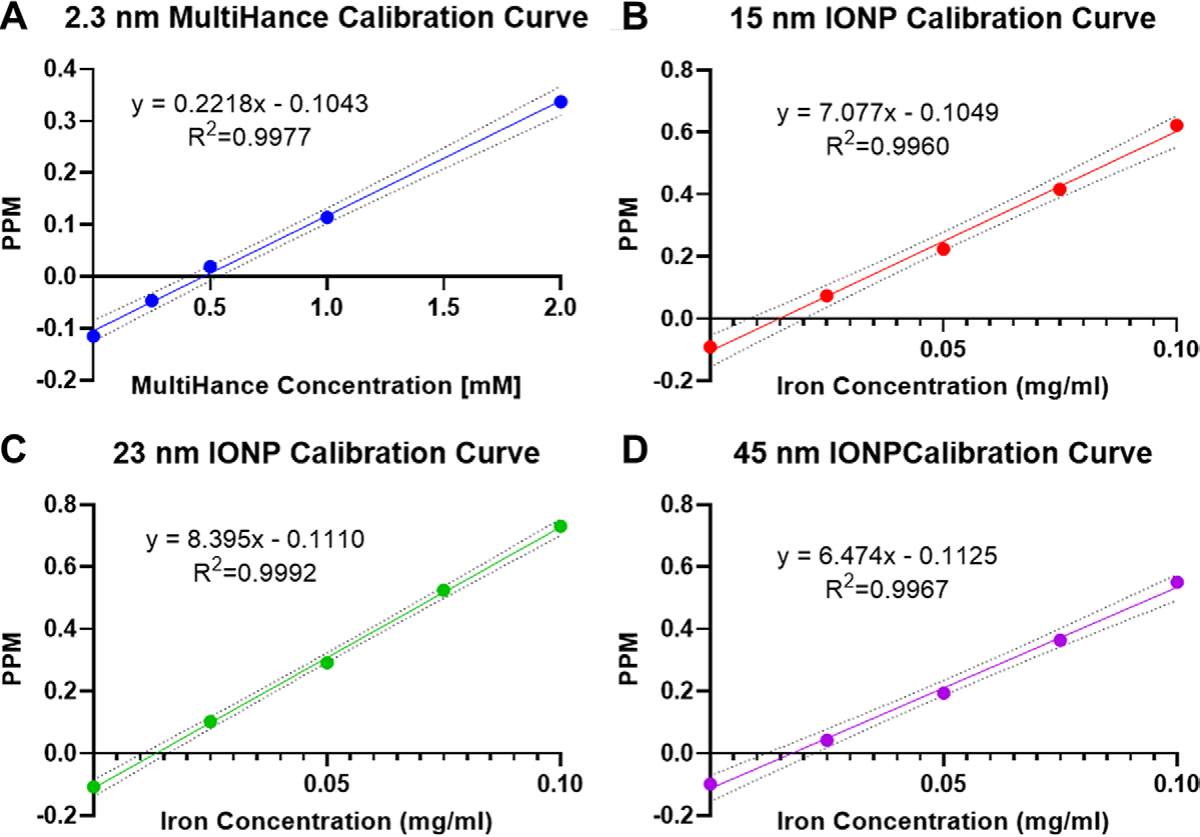
Derivation of contrast agent susceptibilities. **(A)** Susceptibility measurements for MultiHance (2.3 nm) at 0, 0.25, 0.5, 1, and 2 mmol/L (blue circles). **(B–D)** Susceptibility measurements for 15-nm **(B)**, 23-nm **(C)**, and 45-nm **(D)** iron oxide nanoparticles (IONPs) at concentrations of 0, 25, 50, 75, and 100 µg Fe/mL. The slope and y-intercept are reported on each plot, along with the *R*^2^ value, representing the goodness of fit. The dotted gray lines depict the 95% CIs for the line of best fit. In each figure, the *R*^2^ value is high, corresponding to a strong, positive, linear correlation between the contrast agent concentration and the change in the measured magnetic susceptibility of the solution. The data in this figure present the experimentally measured molar or mass magnetic susceptibilities of all contrast agents, derived from the lines of best fit for the contrast agent concentration versus the magnetic susceptibility of the solution.

Before generation of QSM maps in vivo, it was necessary to derive mass susceptibilities for IONPs and the molar susceptibility for MultiHance. This was done by taking the slope of measurements of parts per million changes versus concentration of the respective contrast agents (Fig 2). The mean mass susceptibilities of the 15-, 23-, and 45-nm IONPs were (7.08 ppm · mL)/mg ± 0.26, (8.40 ppm · mL)/mg ± 0.14, and (6.47 ppm · mL)/mg ± 0.22, respectively. The mean molar susceptibility of MultiHance was (0.22 ppm · L)/mmol ± 0.01.

**Figure 3:**
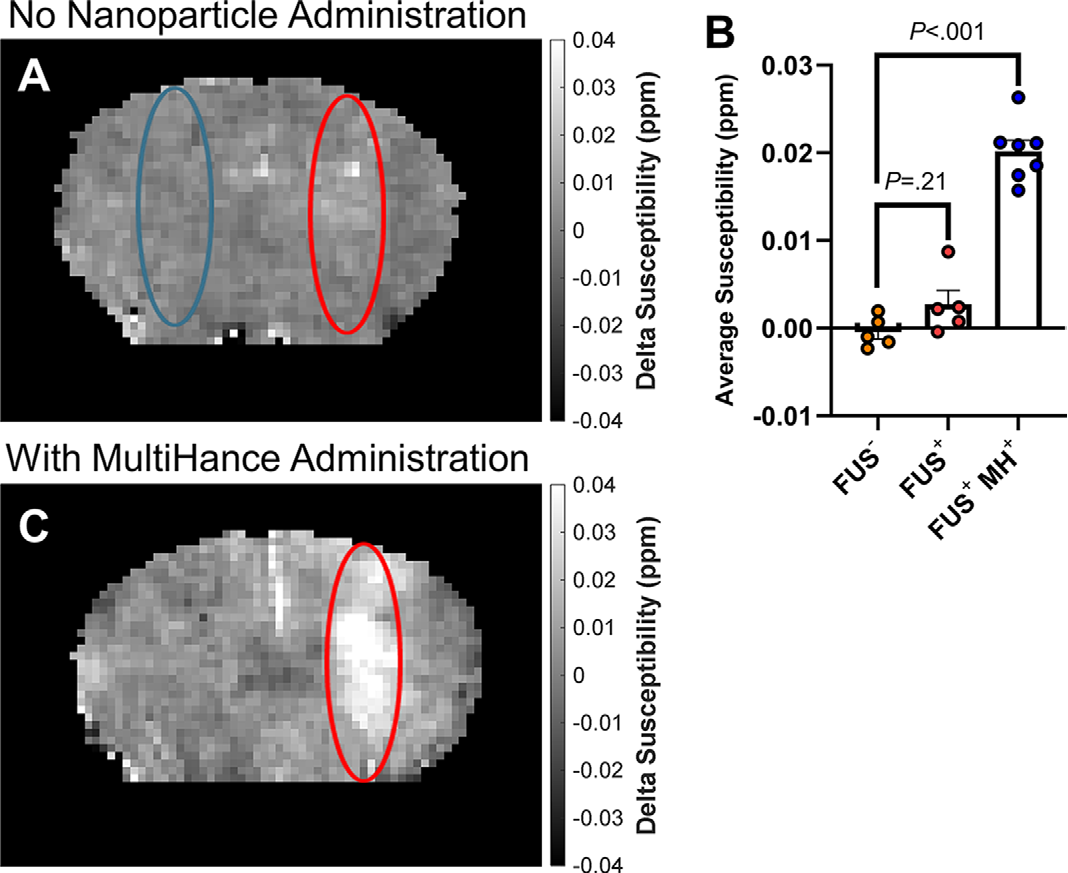
Effect of focused ultrasound (FUS)–mediated blood-brain barrier (BBB) opening in the absence of contrast agent on tissue susceptibility. **(A)** Delta susceptibility map after FUS-mediated BBB disruption but without nanoparticle administration. The red oval designates the FUS treatment region, and the blue oval designates the contralateral region of interest. Darker shading corresponds to a lower delta susceptibility, whereas lighter shading corresponds to a higher delta susceptibility. **(B)** Average susceptibility changes in parts per million are shown for non–FUS-treated (FUS^−^) mouse brain tissue (*n* = 5), FUS-treated (FUS^+^) mouse brain tissue (*n* = 5), and FUS^+ ^tissue with MultiHance delivery (MH^+^) group (*n* = 7). The *P* value for comparison of FUS^−^ with FUS^+^ was .21. The *P* value for comparison of FUS^+^ with MultiHance delivery (MH^+^) versus FUS^− ^was <.001. **(C)** Delta susceptibility map after FUS-mediated BBB disruption with MultiHance delivery. The red oval designates the FUS treatment region. The data in this figure demonstrate that FUS-mediated BBB disruption, in the absence of systemic contrast material administration, produces negligible changes in tissue susceptibility in comparison to treatment-induced changes from contrast agent delivery. Therefore, FUS BBB disruption alone is not biasing delivery measurements.

Next, we verified that FUS-mediated BBB opening alone, under the parameters used in this study and without systemic administration of contrast material, did not significantly affect baseline quantitative susceptibility maps. FUS-mediated BBB opening without contrast agent (*n* = 5) (Fig 3A) resulted in no significant change in tissue susceptibility compared with the contralateral tissue not treated with FUS (hereafter, FUS^−^) (mean delta susceptibility, 0.0027 ppm ± 0.0016 for FUS-treated tissue [hereafter, FUS^+^] and −0.0005 ppm ± 0.0008 for FUS^−^; *P *= .21) (Fig 3B). The FUS^−^ group exhibited small negative biases in susceptibility measurements. To contextualize these susceptibility changes, we compared them to the smallest susceptibility change observed across all treated groups: the MultiHance group (*n* = 7) (Fig 3C) showed an average change of 0.020 ppm ± 0.001. Because this treatment-induced change was 7.4-fold greater than that in the FUS^+^ group, the baseline biases were considered negligible.

**Figure 4:**
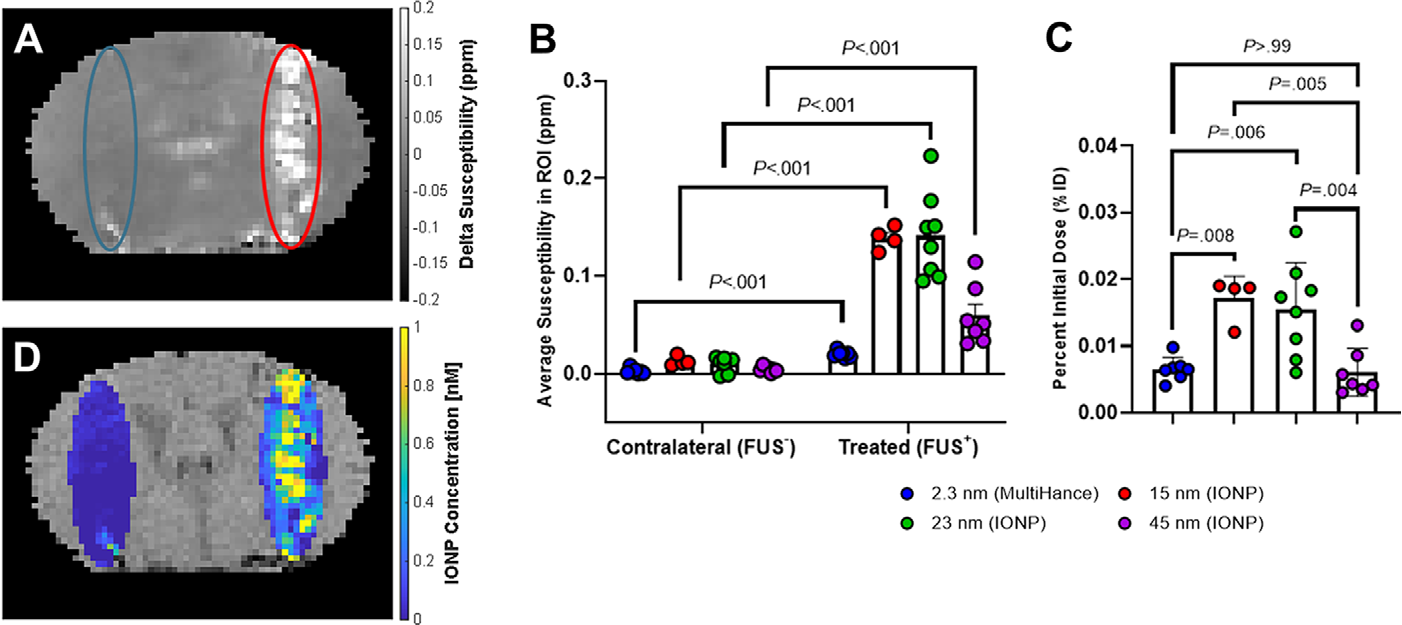
Tissue susceptibility changes in response to focused ultrasound (FUS)–mediated contrast agent delivery and size dependence of delivery. **(A)** Delta susceptibility map after FUS-mediated blood-brain barrier (BBB) disruption and 45-nm iron oxide nanoparticle (IONP) delivery. The red oval designates the FUS treatment region, and the blue oval designates the contralateral region of interest. Darker shading corresponds to a lower delta susceptibility, whereas lighter shading corresponds to a higher delta susceptibility. **(B)** Bar dot plot depicts the average susceptibility changes of the FUS-treated and contralateral brain regions after contrast agent delivery for all four particle size groups (2.3-nm MultiHance [seven mice], 15-nm IONP [four mice], 23-nm IONP [eight mice], and 45-nm IONP [seven mice]). **(C)** Bar dot plot shows the percentage injected dose (%ID) delivered to striatum of mice via FUS-mediated BBB disruption for four particle size groups (2.3-nm MultiHance [*n* = 7], 15-nm IONP [*n* = 4], 23-nm IONP [*n* = 8], and 45-nm IONP [*n* = 7]). The %ID for MultiHance (2.3 nm, 0.007 ± 0.001) was lower than the two intermediate IONPs (15 nm, 0.017 %ID ± 0.002 [95% CI: −0.019 to −0.002; *P *= .008]; 23 nm, 0.015 %ID ± 0.002 [95% CI: −0.016 to −0.002; *P *= .006) but not the 45-nm IONP (0.006 %ID ± 0.001 [95% CI: −0.006 to 0.007; *P* > .99]). **(D)** IONP concentrations derived from susceptibility changes superimposed on the magnitude image. Yellow indicates a nanoparticle concentration of 1 nM, and blue indicates an effective concentration of 0 nM. The data in this figure demonstrate that contrast agent administration alone does not result in meaningful changes in tissue susceptibility in the context of delivery measurements, and a size-dependent relationship exists between FUS-mediated delivery to healthy brain tissue (%ID) and molecular size. FUS^−^ = non–FUS-treated, FUS^+^ = FUS-treated.

Next, alterations in susceptibility in response to contrast agent in the bloodstream, in the absence of FUS, were measured by examining delta susceptibility maps of brain regions contralateral to regions exposed to FUS with contrast agent injection. In FUS-treated regions, mean susceptibility changes were larger (Fig 4A, 4B) and substantially exceeded those on the contralateral side for all four contrast agents (mean ± SEM for MultiHance: *n* = 7, 0.0202 ppm ± 0.0013 vs 0.0027 ppm ± 0.0011, *P* < .001; mean ± SEM for 15-nm IONP: *n* = 4, 0.1386 ppm ± 0.0058 vs 0.0129 ppm ± 0.0025, *P* < .001; mean ± SEM for 23-nm IONP: *n* = 8, 0.1414 ppm ± 0.0155 vs 0.0102 ppm ± 0.0027, *P* < .001; mean ± SEM for 45-nm IONP: *n* = 7, 0.0593 ppm ± 0.0116 vs 0.0044 ppm ± 0.0011, *P* < .001). Although all four contrast agents generated increased susceptibility on the contralateral side, this effect was negligible compared with the treated side, which demonstrated a multifold increase in all cases (fold change in susceptibility between contralateral and FUS-treated regions was as follows: MultiHance, 7.5, *P* < .001; 15-nm IONP, 10.7, *P* < .001; 23-nm IONP, 13.8, *P* < .001; and 45-nm IONP, 13.5, *P* < .001).

### Effect of Therapeutic Size on FUS-mediated Delivery across BBB and BTB

Next, %ID delivered per mouse (Fig 4C) was calculated via molar concentrations (Fig 4D) derived from delta susceptibility maps. The mean %ID for MultiHance (2.3 nm, 0.007 [0.7 nmol/10 000.0 nmol] ± 0.001) was lower than the two intermediate IONPs (15 nm: 0.017 [0.59 pmol/3450 pmol] ± 0.002, 95% CI: −0.019 to −0.002, *P* = .008; 23 nm: 0.015 [0.066 pmol/430 pmol] ± 0.002, 95% CI: −0.016 to −0.002, *P* = .006) but not the 45-nm IONP (0.006 [1.03 fmol/17 000.00 fmol] ± .001, 95% CI: −0.006 to 0.007, *P* > .99). In addition, the %IDs of the 15-nm and 23-nm IONPs were greater than that of the 45-nm IONP (15 nm: 95% CI: 0.003 to 0.019 [*P* = .005]; 23 nm: 95% CI: 0.003 to 0.016 [*P* = .004]) but did not differ between each other (15 nm vs 23 nm; 95% CI: −0.006 to 0.009 [*P* = .94]).

**Figure 5:**
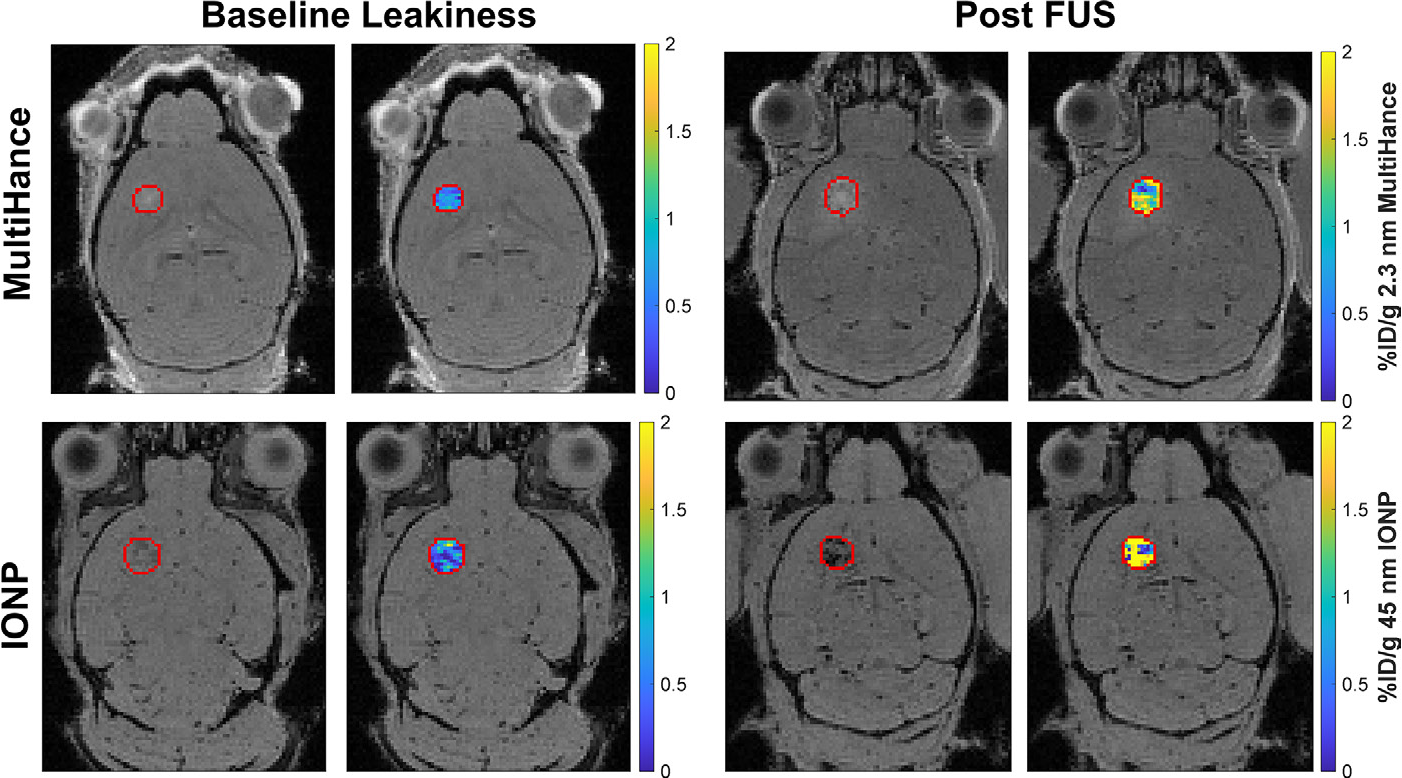
Representative images in mice with GL261 tumor-bearing brains that underwent MRI with superimposed concentration maps for MultiHance and 45-nm iron oxide nanoparticle (IONPs). Baseline and focused US (FUS)–treated conditions for 8–11-week-old female glioma-bearing mice that received MultiHance (*n* = 2) or 45-nm IONP (*n* = 2) administration are shown (left and right, respectively). Each condition includes two paired images: the anatomic image with the tumor region of interest outlined in red (left) and the corresponding image with a concentration map superimposed over the tumor region (right). The top row shows representative images for MultiHance administration, and the bottom row shows representative images for 45-nm IONP administration. For both sets of images, yellow represents a voxel percentage injected dose per gram (%ID/g) value of 2 or greater, and blue represents an effective value of 0. The images in this figure demonstrate that quantitative susceptibility mapping can measure both the amount and spatial distribution of MultiHance and a 45-nm IONP after FUS-mediated blood-tumor barrier disruption.

**Figure 6:**
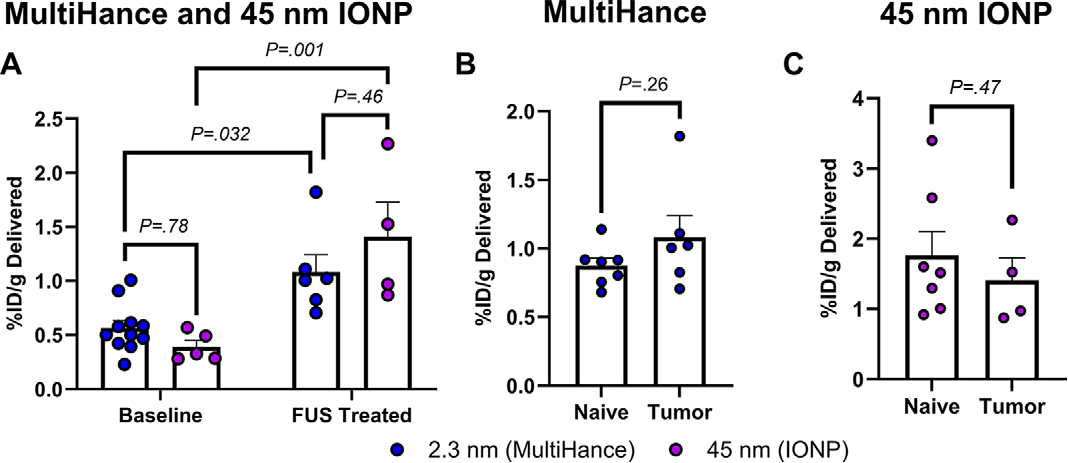
Direct comparison of focused ultrasound (FUS)–mediated, size-dependent therapeutic delivery of MultiHance and a 45-nm iron oxide nanoparticle (IONP) to naive brain and GL261 brain tumors in mice. **(A)** Baseline tumor leakiness to MultiHance (*n* = 11; percentage injected dose per gram [%ID/g], 0.56 ± 0.07) showed no difference compared with that of a 45-nm IONP (*n* = 5; 0.39 ± 0.06 [95% CI: −0.34 to 0.68; *P *= .78]). FUS enhanced delivery of both contrast agents (MultiHance: *n* = 11, 0.56 ± 0.07 vs *n* = 6, 1.08 ± 0.16 [95% CI: −1.00 to −0.04; *P *= .032]; 45-nm IONP: *n* = 5, 0.39 ± 0.06 vs *n* = 4, 1.41 ± 0.32 [95% CI: −1.65 to −0.38; *P *= .001]).** (B)** Comparison of the FUS-mediated delivery of MultiHance across the healthy blood-brain barrier (BBB) and the blood-tumor barrier (BTB) (*n* = 7, 0.87 ± 0.06 vs *n* = 6, 1.08 ± 0.16 [95% CI: −0.20 to 0.62; *P *= .26]). **(C)** Comparison of the FUS-mediated delivery of a 45-nm IONP across the healthy BBB and the BTB (*n* = 7, 1.76 ± 0.34 vs *n* = 4, 1.41 ± 0.32 [95% CI: −1.43 to 0.72; *P *= .47]). The data in this figure show that glioma delivery, both at baseline and after FUS, did not differ between a 2.3- and 45-nm particle and that the FUS-mediated delivery to gliomas of both particles was comparable to that to healthy brain.

Subsequently, FUS was applied to mouse glioma GL261 brain tumors to deliver 45-nm IONPs (*n* = 4) and MultiHance (*n* = 6) using parameters identical to those used in the naive brain experiments. Pre- and post-FUS susceptibility changes in brain tumors were converted to the %ID/g delivery metric (Fig 5). At baseline, tumor leakiness to MultiHance, reported as mean %ID/g ± SEM (*n* = 11, 0.56 [0.22 pmol/39.56 pmol · g] ± 0.07) was no different than leakiness to 45-nm IONPs (*n* = 5, 0.39 [0.26 amol/67.25 amol · g] ± 0.06, 95% CI: −0.34, 0.68, *P *= .78) (Fig 6A). Furthermore, FUS enhanced delivery of both contrast agents (MultiHance: 0.56 [0.22 pmol/39.56 pmol · g] ± 0.07 vs 1.08 [0.43 pmol/39.56 pmol · g] ± 0.16, 95% CI: −1.00 to −0.04, *P *= .032; 45-nm IONP: 0.39 [0.26 amol/67.25 amol · g] ± 0.06 vs 1.41 [0.95 amol/67.25 amol · g] ± 0.32, 95% CI: −1.65 to −0.38, *P *= .001) (Fig 6A). This equated to a 1.9-fold increase over baseline for MultiHance and 3.6-fold increase for IONP, but comparisons of fold change over mean baseline demonstrated no size dependencies at this timepoint (*P *= .06) (Fig S1).

### Direct Comparison of FUS-mediated, Size-dependent, Therapeutic Delivery to Naive Brain versus Brain Tumors

Next, the effect of dysregulated brain tumor vascular structure, in combination with high interstitial tumor pressure, on FUS-mediated delivery was examined in mice with brain tumors and compared with naive brain. With use of identical dosages and FUS parameters, FUS-mediated delivery of MultiHance (2.3 nm) and a 45-nm IONP to GL261 tumors (ie, trans-BTB), measured by %ID/g delivered, was similar to delivery to naive brain (ie, trans-BBB) (MultiHance: 1.08 [0.43 pmol/39.56 pmol · g] ± 0.16 vs 0.87 [0.34 pmol/39.56 pmol · g] ± 0.06, 95% CI: −0.20 to 0.62, *P* = .26 [Fig 6B]; 45-nm: 1.41 [0.95 amol/67.25 amol · g] ± 0.32 vs 1.76 [1.18 amol/67.25 amol · g] ± 0.34, 95% CI: −1.43 to 0.72, *P* = .47 [Fig 6C]).

## Discussion

Critical yet previously underexplored topics in the field of focused ultrasound (FUS)–mediated therapeutic delivery to the brain include *(a)* the precise influence of therapeutic size on absolute delivery (eg, units of millimolars, percentage injected dose [%ID], or %ID per gram) and *(b)* whether dysregulated tumor microenvironment affects the absolute delivery of various size agents when compared with naive brain. To address these knowledge gaps, we proposed the use of quantitative susceptibility mapping (QSM) to measure the delivery of contrast agents of therapeutically relevant sizes in the context of FUS-mediated blood-brain barrier (BBB) and blood-tumor barrier disruption. Our QSM analysis revealed two key findings: *(a)* FUS-mediated nanoparticle delivery across the BBB to naive brain is size-dependent, with %ID delivered increasing 2.6-fold from 2.3 nm to 15 nm (*P* = .008) but decreasing 2.5-fold from 23 nm to 45 nm (*P *= .004), and *(b)* the dysregulated tumor microenvironment does not appear to significantly affect delivery for small or larger particles, as evidenced by similar %ID per gram for both MultiHance (2.3 nm) and 45-nm IONPs in tumor versus naive brain (*P * = .26 and .47, respectively).

For naive brain, an initial increase in %ID delivered from the 2.3-nm to 15-nm and 23-nm particles was surprising when compared with previous studies showing that increasing the size of contrast agents negatively affects FUS-mediated delivery (3,4,27–29). Discrepancies between our results and those of previous studies could be attributed, at least in part, to FUS and microbubble differences. Indeed, it is known that FUS parameters (eg, pressure, frequency, pulse, and treatment duration) and microbubble characteristics (eg, size and composition) significantly impact delivery across the BBB (7,10,27,30). To enhance clinical relevance, we deployed an acoustic emissions feedback control system that capped and modulated FUS pressure during treatment, similar to approaches used in clinical studies (31). It is possible this control system yields BBB opening outcomes that differ from those reported in previous studies. Yet, we believe the more likely explanation is that QSM is revealing previously unappreciated relationships between trans-BBB delivery with FUS and particle size. This is because, unlike previous fluorescence studies, our QSM approach allowed us to normalize quantitative absolute mass delivery measurements to injected dose.

Our findings underscore the nuanced interplay between transport resistance across the barrier and particle clearance kinetics (10,32). Differences in blood half-lives in mice between MultiHance and IONPs provide context: Gadolinium-based contrast agents such as MultiHance clear rapidly from the vasculature (blood half-life, approximately 2 minutes), whereas ferumoxytol, a proxy for IONPs, exhibits a markedly longer intravascular half-life (approximately 45 minutes) (4,33,34). This variance in clearance kinetics could explain the bell-shaped relationship between %ID and hydrodynamic diameter. Consequently, there may be an optimal particle size between 2.3 nm and 45 nm, balancing rapid clearance of smaller molecules against the increased transport restrictions of larger particles—a hypothesis supported by previous bulk delivery measurements of gold nanoparticles to murine brains (10).

It is crucial to recognize that our measurements reflect a complex combination of BBB transport, clearance kinetics, and barrier closure dynamics, rather than solely barrier transport efficiency. Accordingly, the differences in the pharmacokinetic profiles of the contrast agents should be considered in interpreting these results because delivery was evaluated at a single time point (approximately 25 minutes after barrier opening). This time point likely corresponds to different stages of each agent’s pharmacokinetic profile. Because delivery is a dynamic process, the size-dependent relationships observed here may differ at earlier or later time points. Nonetheless, these findings provide critical insight for the clinical implementation of FUS-mediated BBB delivery, demonstrating that therapeutic size significantly influences delivery and should be considered when selecting treatments for individual patients.

QSM delivery (%ID/g) measurements from GL261-Luc2 tumors revealed a lack of size-dependent effects on both baseline leakiness and FUS-mediated delivery. The similar baseline leakiness between MultiHance and 45-nm IONPs was surprising given the known differences in vascular permeability with respect to particle size within tumors. However, an unpaired *t* test comparison of these groups outside of the two-way analysis of variance revealed a trend toward MultiHance possessing significantly higher baseline delivery (*P* = .07) (Fig S2). Such a result is more consistent with conventional ideas of tumor permeability and particle size, but our quantitative approach to delivery assessment may also reveal a weaker relationship than previously thought (35,36). After FUS, the delivery of MultiHance was similar to that of the 45-nm IONPs, both in absolute terms and in relative increase over baseline (Figs 6A, S1). Although initially unexpected, this convergence is consistent with observations in naive brain tissue, where no significant difference in baseline delivery between the two agents was observed. These findings highlight the therapeutic potential of FUS for delivering larger agents, such as gene therapies and nanoparticles, into tumors.

Intraparticle comparisons of delivery across the healthy BBB (naive mice) and the BTB (GL261-Luc2 tumors) for MultiHance and a 45-nm IONP revealed a lack of difference for both molecules. Given the dysregulated tumor vasculature that forms the BTB, we anticipated a significant effect on FUS-mediated delivery in both cases. However, because this quantitative comparison had not been previously made, the expected directionality of this effect relative to the healthy BBB remained speculative. The parallels between naive brain and tumor are further reinforced by similar delivery for MultiHance and the 45-nm IONP in both instances. This finding suggests that dysregulated tumor vasculature and microenvironment may affect FUS-mediated delivery less than previously anticipated.

All four phantom measurements exhibited a negative susceptibility offset for saline while maintaining strong linearity with increasing contrast agent concentration (Fig 2). We attribute this offset to diamagnetic contributions from the plastic vials, which likely introduced a uniform negative shift across all samples and therefore did not affect the accuracy of mass or molar susceptibilities derived from the slope. The measured susceptibility of MultiHance (0.222 [ppm · L/mmol]) was lower than some commonly cited values for gadolinium measured with Magnevist (0.326 [ppm · L/mmol]) but was consistent with another measured with gadofosveset trisodium (0.22 [ppm · L/mmol]) (37,38). Such discrepancies could be tied to phantom orientation. In our case, vials were imaged perpendicular to the main magnetic field, a geometry shown to reduce measured susceptibility (39,40). This orientation was chosen to better mimic the columnar distribution of contrast agent in the brain after FUS (Fig 4A). Differences in acquisition parameters and QSM processing steps may also contribute to calibration discrepancies, which is why we chose to calibrate all nanoparticles using the same analysis pipeline used for in vivo measurements (40).

This study had limitations. Delivery was assessed at a single time point (approximately 25 minutes after FUS), providing only a snapshot of a dynamic process influenced by transport and clearance kinetics. Particles larger than 45 nm were not tested, although size effects likely extend beyond this range. Verification of IONP concentrations using complementary imaging (eg, PET/MRI with radiolabeled IONPs) or histologic staining could further validate QSM measurements. In addition, QSM assumes a static, uniform main field; residual IONPs, microbubbles, or proximity to the metallic FUS transducer may have introduced susceptibility artifacts and increased reliance on background field removal (projection onto dipole fields). Nonetheless, these factors did not appear to affect the repeatability or reproducibility of quantitative susceptibility maps across subjects or sessions. Finally, the delta susceptibility maps within tumors occasionally exhibited negative values at the dorsal and ventral ends of the region of interest, near the skull. These effects likely reflect limitations in background field removal or dipole inversion. However, these negative values were retained within the region of interest to avoid biasing the resulting bulk %ID/g delivery measurements.

In conclusion, quantitative susceptibility mapping after MRI and focused ultrasound (FUS) revealed that FUS-mediated delivery of contrast agents of therapeutically relevant sizes to naive murine brain varied with contrast agent size, and delivery to gliomas was similar to naive brain despite the presence of dysregulated glioma vasculature and the tumor microenvironment. Future work should assess multiple time points and larger molecules and directly correlate these findings with therapeutic outcomes. Examining the influence of particle surface chemistry on delivery could clarify which physical properties best synergize with FUS-mediated transport.

## Supplemental Files

Appendices S1-S2, Figures S1-S2

Conflicts of Interest
