# Supplementary material for: Quantitative Susceptibility Mapping Shows Size-dependent Focused Ultrasound–mediated Therapeutic Delivery to Naive Brain and Gliomas in Mice
Source: Radiology. 2026 Jul 21;320(1):e251005. doi: 10.1148/radiol.251005 (PMC13421230; doi:10.1148/radiol.251005)
Supplement: Appendices S1-S2, Figures S1-S2 [file ry251005supp.pdf]

Supplemental materials undergo peer review but are not copyedited, and may include typographical errors.

©RSNA, 2026
10.1148/radiol.251005

Appendix S1

Luciferase-transduced mouse glioma GL261 cells (GL261-Luc2) ($1x10^5$ cells per 2 µL) were suspended in sterile phosphate-buffered saline then implanted into the right striatum of twenty three 8–10-week-old female C57BL/6 mice (The Jackson Laboratory, Bar Harbor, ME). A mechanically controlled rate of 0.5 µL/min with a 10 µL Hamilton syringe and micropump (UltraMicroPump, World Precision Instruments) were used to inject the GL261-Luc2 cells at ~2.0 mm lateral from the sagittal suture, 0.5 mm anterior of bregma, and 3 mm below the dura.

Supplemental materials undergo peer review but are not copyedited, and may include typographical errors.

Appendix S2

For the naïve brain studies, regions of interest were drawn [MRH; 4 years of experience] based on IONP or gadolinium concentration maps derived from pre- and post-FUS susceptibility changes to obtain %ID data. Regions of interest were carefully drawn around voxels in the target location with visually enhanced concentration values while avoiding the ventricles. To conduct contralateral analysis, regions of interest were drawn in the same approximate anatomical location but mirrored across the midline. For the FUS-positive, nanoparticle-negative control, regions of interest of similar shape and size to those in the MultiHance group were drawn at the treatment location. For tumor-bearing mice, regions of interest were drawn based on regions of enhanced susceptibility in pre-FUS QS maps, with magnitude images and knowledge of tumor implantation site serving as additional guides for localization. Regions of interest were drawn to fully encompass the tumor volume.

Supplemental materials undergo peer review but are not copyedited, and may include typographical errors.

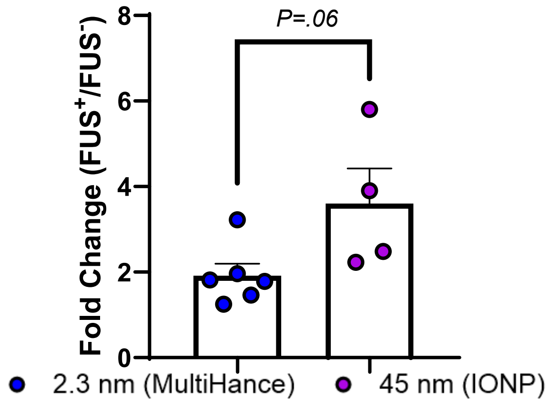

Figure S1. Comparison of Fold Change in Delivery Over Baseline Introduced by Focused Ultrasound in Gliomas for MultiHance and a 45 nm Iron Oxide Nanoparticle. Fold change over baseline generated by focused ultrasound (FUS) in gliomas. Fold change was calculated by using the average %ID/g of the baseline group for each respective particle.

Supplemental materials undergo peer review but are not copyedited, and may include typographical errors.

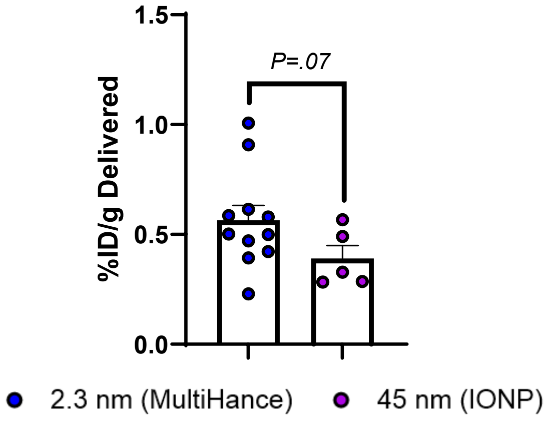

Figure S2. Comparison of Baseline Leakiness in Gliomas for MultiHance and a 45 nm Iron Oxide Nanoparticle. Baseline tumor leakiness to MultiHance, (n=11, %ID/g: 0.56 ± 0.07) showed no difference compared to that of a 45 nm iron oxide nanoparticle (IONP) (n=5, 0.39 ± 0.06, 95% CI: -0.37 to 0.02, *P*=.07).
